# Interference of TiO_2_ in Fricke Dosimetry: Adsorption Mechanisms and Dose Measurement Challenges under Gamma Irradiation

**DOI:** 10.1002/open.202500524

**Published:** 2026-03-29

**Authors:** William Villacis, Roque Santos, María Natalia Piol, Paul Vargas‐Jentzsch, Cristina Vázquez

**Affiliations:** ^1^ Escuela Politécnica Nacional Facultad de Ingeniería Química y Agroindustria Departamento de Ciencias Nucleares Centro de Irradiación Quito Ecuador; ^2^ Facultad de Ingeniería Departamento de Química Universidad de Buenos Aires Buenos Aires Argentina

**Keywords:** adsorption, dosimetry, iron compounds, radiation chemistry, titanium

## Abstract

This review examines the interaction between Fricke dosimetry and TiO_2_ under gamma irradiation, highlighting the potential interference of TiO_2_ in standard dosimetric systems, particularly when this semiconductor is employed as a radiosensitizer (or, more accurately, a radiocatalyzer). The classical Fricke dosimetry, based on the radiolytic oxidation of Fe^2+^ to Fe^3+^, may be influenced by TiO_2_ through adsorption, therefore compromising dose accuracy. A recopilation of information on adsorption phenomena from both kinetic and equilibrium perspectives, applying classical and advanced models to characterize the retention of Fe^2+^ and Fe^3+^ on TiO_2_ surfaces, was performed. Particular attention is given to multicomponent systems that reflect the competitive nature of adsorption under Fricke conditions (acidic medium). We discuss how these interactions affect dosimetric responses, potentially leading to the underestimation of the radiation absorbed doses. Although the increasing use of TiO_2_ in radiocatalysis and advanced oxidation processes, standardized protocols for chemical dosimetry in such conditions are still missing. Research gaps include insufficient adsorption mechanistic studies, limited application of multicomponent models, and scarce integration of adsorption parameters into dosimetric assessments. Finally, methodological approaches to enhance dosimetric reliability in the presence of TiO_2_ were proposed, thereby contributing to the development of accurate and reproducible systems for several applications.

## Graphical abstract

The presence of TiO_2_ in Fricke dosimetric systems introduces complex interferences under gamma irradiation. Adsorption of Fe^2+^/Fe^3+^ ions, coupled with radiolytic and surface‐mediated processes, alters redox equilibria and apparent absorbed dose values, highlighting critical challenges in chemical dosimetry applied to heterogeneous radiolytic environments.

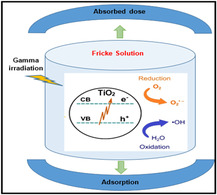

## 1 Introduction

Ionizing radiation, encompassing both electromagnetic (e.g., gamma rays and X‐rays) and particulate forms (e.g., alpha and beta particles), has been extensively utilized in various industrial and medical applications due to its ability to induce chemical and physical changes in matter [1, 2, 3]. Accurate measurement of the absorbed radiation dose is essential to ensure both the efficacy and safety of these applications [4, 5, 6, 7]. Broadly, two main approaches exist for quantifying absorbed dose within a medium: physical dosimeters and chemical dosimeters. Physical dosimeters rely on detectable physical changes in materials, such as alanine pellets or thermoluminescence dosimeters, whereas chemical dosimeters are based on radiation‐induced chemical transformations, typically monitored through variations in the concentration of specific chemical species [8, 9]. Among the chemical dosimeters available, the Fricke dosimeter, based on the oxidation of ferrous ions (Fe^2+^) to ferric ions (Fe^3+^) in an acidic aqueous solution, has been widely recognized for its reliability and precision in quantifying absorbed doses in water‐equivalent media [10, 11].

Parallel to these developments, titanium dioxide (TiO_2_), a semiconductor material, has attracted growing attention owing to its photocatalytic and radiocatalytic properties, particularly in the context of environmental remediation [12]. Upon exposure to ionizing radiation, TiO_2_ generates electron–hole pairs that promote the formation of reactive oxygen species (ROS) capable of degrading diverse organic pollutants [12, 13]. Beyond its catalytic role, TiO_2_ also exhibits notable adsorption properties, enabling interactions with ionic species in solution [14]. This dual behavior becomes especially relevant when TiO_2_ is present in systems where chemical dosimetry, such as the Fricke solution, is employed.

Although acidic environments generally hinder the adsorption of cations such as Fe^2+^ and Fe^3+^ on TiO_2_, due to the positive surface charge below its point of zero charge (pHpzc ≈ 6 – 6.6) [15, 16] several mechanisms make this interaction plausible. Surface complexation, partial hydrolysis of ferric ions [17], and the strong affinity of TiO_2_ for multivalent metals can promote adsorption even at low pH. Moreover, under irradiation conditions, transient reactive species may further enhance these interactions [18], providing a rational basis for exploring adsorption phenomena within the acidic Fricke system.

The convergence of these two domains—radiation dosimetry and photocatalytic adsorption—creates both challenges and opportunities. When the Fricke dosimeter is applied in media containing TiO_2_, there is a potential for Fe^2+^ and Fe^3+^ ions to adsorb onto the TiO_2_ surface. Such interactions may disturb the ionic equilibrium and, consequently, affect the accuracy of dosimetric measurements. Addressing this issue requires an understanding of the kinetics and equilibrium mechanisms governing ion adsorption, particularly under acidic conditions that characterize the Fricke system [19].

In this review, we critically analyze recent advances in chemical analysis concerning the Fricke dosimeter and TiO_2_ adsorption phenomena. We discuss the principles of radiation dosimetry using the Fricke solution, highlight the photocatalytic and adsorptive behavior of TiO_2_, and examine the kinetic and isotherm models relevant to Fe ion adsorption. By elucidating the interplay between these components, this work aims to provide insights that support accurate dose determination in complex systems where TiO_2_ is present.

## 2 Radiation Dosimetry and Fricke Solution

Radiation dosimetry is a crucial discipline for quantifying the amount of energy absorbed by matter when exposed to ionizing radiation [20]. Among the standard methods used, Fricke dosimetry stands out as a well‐established, precise, and reproducible chemical technique. It relies on the radiation‐induced oxidation of Fe^2+^ to Fe^3+^ in an acidic aqueous solution saturated with oxygen [21]. This oxidation process is initiated by reactive species generated during the radiolysis of water, primarily hydroxyl radicals (•OH), hydrogen atoms (•H), and hydrated electrons (e^−^_aq_) [22]. These radicals react with Fe^2+^ in an acidic aqueous solution (typically 0.4 M sulfuric acid), leading to the formation of Fe^3+^ [23].

The relevant reactions involved in the dosimetric response are shown in Equations (1)–(4) [22, 23].

(1) $$\begin{array}{c} {\mathrm{Fe}}^{2+}+•\mathrm{OH}\;\to \;{\mathrm{Fe}}^{3+}+{\mathrm{OH}}^{-}\\ H+O_{2}\;\to \;{\mathrm{HO}}_{2} \end{array}$$

(2) $$\begin{array}{c} {\mathrm{Fe}}^{2+}+{\mathrm{HO}}_{2}\;\to \;{\mathrm{Fe}}^{3+}+{{\mathrm{HO}}_{2}}^{-}\\ {{\mathrm{HO}}_{2}}^{-}+H^{+}\;\to \;H_{2}O_{2} \end{array}$$

(3) $${\mathrm{Fe}}^{2+}+H_{2}O_{2}\;\to \;{\mathrm{Fe}}^{3+}+•\mathrm{OH}+{\mathrm{OH}}^{-}$$

(4) $${\mathrm{Fe}}^{2+}+•\mathrm{OH}\;\to \;{\mathrm{Fe}}^{3+}+{\mathrm{OH}}^{-}$$

The solution must be maintained under strongly acidic conditions (pH < 3), typically by using 0.4 M sulfuric acid. This acidity is essential to stabilize both Fe^2+^ and Fe^3+^ species and to suppress undesired side reactions such as hydrolysis, radical recombination, or disproportionation that would otherwise interfere with the dosimetric response [24, 25].

A typical Fricke solution is composed of 1 mM ferrous ammonium sulfate, 1 mM sodium chloride, and 0.4 M sulfuric acid. The high ionic strength and strongly acidic environment maintain chemical stability and enhance the selectivity of the Fe^2+^ oxidation. Upon irradiation, the concentration of Fe^3+^ increases and is quantified spectrophotometrically by measuring the absorbance at 304 nm, following the Beer–Lambert law [26, 27, 28, 29, 30].

The absorbed dose (D, in Gy) is calculated from the change in absorbance (Δ*A*) according to the following Equation (5) [20, 21, 31, 32].

(5) $$D(\mathrm{Gy})=\frac{\text{Δ}A*N_{A}}{G\left({\mathrm{Fe}}^{3+}\right)*\varepsilon *d*\rho *f_{1}}$$

The factors considered in Equation (5) are listed in Table 1, which are required to calculate the absorbed dose. The names of the factors as well as important details related to them are also presented.

**TABLE 1:** Factors used in the equation for the determination of the absorbed dose by Fricke dosimetry.

Parameter	Name	Value	Observation	Reference
Δ*A*	Change in absorbance or relative optical density	—	Absorbance at a wavelength of 304 nm (maximum absorption of Fe^3+^) of irradiated and nonirradiated solutions	[20, 21, 26, 31, 32]
*N*_A_	Avogadro's number	6.022 × 10^23^ mol^−1^		[20, 31, 32]
*ρ*	Density of the solution	1.024 × 10^3^ kgm^−3^	Temperature = 25°C	[10, 20, 26, 32]
G(Fe^3+^)	Chemical yield of irradiation energy	9.74 × 10^17^ J^−1^	Electrons or photons with energies ranging from 0.5 to 16 MeV at absorbed dose rates < 2 × 10^7^ Gy/s	[20, 21, 31, 33]
*ε*	Molar extinction coefficient	219.5 m^2^*mol^−1^	At a wavelength of 304 nm and a temperature of 25°C.	[10, 20, 32, 33]
*d*	Optical pathway	0.01 m	Length of the optical path in quartz cells	[10, 20, 31, 33]

The G(Fe^3+^) factor, also named G‐value, provides an index of the linear response of the dosimeter. It is defined as the number of ferric ions produced per 100 eV of the radiation energy absorbed by the Fricke solution [20, 31].

Since temperature affects both the density and the molar extinction coefficient of the solution, a correction factor (*f*_1_) is applied when absorbance is measured at a temperature other than 25°C. The factor *f*_1_ was determined using Equation (6), where *T*_l_ represents the temperature of the solution during the absorbance measurement (°C) [32].

(6) $$f_{1}=1+0,0069(T_{l}-25)$$

Advantages of Fricke dosimetry include its high accuracy, linear dose response over a wide range (typically 20 Gy to several kGy), chemical stability under proper conditions, and traceability to international dosimetric standards^.^ [9, 5] Furthermore, it is simple to prepare and analyze using conventional UV–Vis spectrophotometry.

However, limitations also exist. The Fricke solution is sensitive to impurities, requires strict oxygen saturation, and is only suitable for homogeneous radiation fields. It is incompatible with high dose rates or pulsed radiation due to radical recombination effects. Moreover, the requirement for a strongly acidic medium (pH < 3) may limit its applicability in some experimental setups and precludes in vivo applications, [34, 35, 24].

In conclusion, the Fricke dosimeter remains a gold standard in chemical dosimetry, offering a balance of simplicity, accuracy, and well‐characterized response, provided its operational constraints are properly managed.

## 3 Titanium Dioxide in Radiolytic Systems

TiO_2_ has been extensively investigated for its unique physicochemical properties, particularly its stability, nontoxicity, and strong oxidizing potential under UV–Vis light irradiation [35, 36]. Structurally, TiO_2_ exists in three main crystalline forms—anatase, rutile, and brookite—with anatase generally considered the most photoactive phase due to its higher surface area and superior charge separation efficiency [37, 38]. These structural differences, along with the material's wide bandgap (∼3.2 eV for anatase and ∼3.0 eV for rutile), render TiO_2_ an efficient photocatalyst under ultraviolet (UV) [39, 12, 40, 41]. This semiconductor character underlies the initiation of redox reactions when TiO_2_ is exposed to energy sources sufficient to excite electrons from the valence band to the conduction band, thereby generating electron–hole pairs (e^−^/h^+^) [12, 42, 43].

Upon UV–Vis light radiation exposure, these charge carriers migrate to the surface where they interact with adsorbed species. The photogenerated holes (h^+^) can oxidize water or hydroxide ions to produce hydroxyl radicals (•OH), while the conduction band electrons (e^−^) can reduce molecular oxygen to generate superoxide radicals (O_2_•^−^) [39, 36, 44, 45]. These ROS are key intermediates in the degradation of organic pollutants and contribute to mineralization pathways, especially in aqueous environments. The overall mechanism, often referred to as heterogeneous photocatalysis, is strongly influenced by several factors, including crystallinity, surface area, particle size, and the presence of defects or dopants, which affect the recombination rates of the e^−^/h^+^ pairs and the lifetime of reactive intermediates [46, 47].

When TiO_2_ is applied in radiolytic systems—such as those involving gamma or electron beam irradiation—it acts synergistically with the products of water radiolysis. Ionizing radiation decomposes water molecules, generating highly reactive species such as hydroxyl radicals (•OH), hydrated electrons (e^−^_aq_), and hydrogen atoms (H•). TiO_2_ can interact with these species in multiple ways: it facilitates electron–hole transfer at its surface, stabilizes transient radicals, and promotes redox reactions that enhance the degradation of surrounding solutes. This interplay between TiO_2_ and primary radiolysis products provides the mechanistic basis for its role as a radiosensitizer in aqueous systems. Since TiO_2_ is a semiconductor, gamma rays photons will have enough energy to promote electron from the conduction of the valence band to the conduction band, and thus producing electron hole pairs, similar to those formed under UV–vis light [39, 48].

It is widely assumed that the photo‐induced formation mechanism of the electron‐hole pair in the TiO_2_ photocatalyst is governed by several chemical reactions. Among them, the following equations describes the main ones [44].

(7) $$\mathrm{Photo}‐\mathrm{excitation}:{\mathrm{TiO}}_{2}+hv\;\to \;e^{-}+h^{+}$$

(8) $$\mathrm{Oxidationofhydroxyls}:{\mathrm{OH}}^{-}+h^{+}\;\to \;•\mathrm{OH}$$

(9) $$\mathrm{Photodegradation}\;\mathrm{by}\;•\mathrm{OH}:\;R-H+•\mathrm{OH}\;\to \;R\prime •+H_{2}O$$

We must pay special attention to Equation (7) which describes the creation of an electron/hole pair by the action of a photon that carries an amount of energy equals to *hv*, where *h* is the plank constant and *v* is the wavelength of the photon. For this reaction to take place, it is necessary that the energy *hv* is higher than the binding energy of the electron to the atom. Ionizing radiation will have much higher energy that this threshold, but more importantly, ionizing radiation will create secondary electrons that will interact with the electrons of the valence band and will promote them to the conduction band. So, gamma rays will create a higher number of electron/holes in the medium. This is the mechanism for radiocatalysis [49, 50, 51].

TiO_2_ surfaces can interact with the other species created during photon or radiation interaction, particularly by capturing electrons and enhancing the yield of oxidizing radicals. This dual functionality, combining semiconductor photocatalysis and radiation‐induced radical chemistry, makes TiO_2_ an attractive material for advanced oxidation processes aimed at degrading persistent pollutants in water, including pharmaceutical compounds and antibiotics [52, 53].

In radiolytic systems, as we briefly discussed earlier, TiO_2_ exhibits a distinct behavior when exposed to ionizing radiation such as gamma rays or electron beams, compared to its well‐known photocatalytic activity under ultraviolet (UV) light. Although the fundamental mechanism of electron–hole pair generation remains analogous, the source of energy and the subsequent physicochemical context diverge notably. Upon exposure to ionizing radiation, high‐energy photons or particles interact with water molecules and TiO_2_ crystals, resulting in the formation of reactive intermediates. These interactions generate conduction‐band electrons (e^−^_cb_) and valence‐band holes (h^+^_vb_) within the TiO_2_ structure. The presence of radiation further leads to the radiolysis of water, producing species such as hydrated electrons (e^−^_aq_), hydroxyl radicals (•OH), and hydrogen atoms (•H). The synergy between TiO_2_ and water radiolysis products amplifies the redox activity in solution. The gamma‐generated holes in TiO_2_ may oxidize water or hydroxide ions to •OH, while the electrons can reduce protons to produce H_2_ or participate in the reduction of oxygen to superoxide radicals (O_2_•^−^), similar to classical photocatalysis mechanisms [49, 42, 54].

Nonetheless, under ionizing radiation, TiO_2_ does not rely on direct photon absorption as in UV‐photocatalysis. Instead, it acts as an electron mediator or sensitizer, enhancing the yield and selectivity of ROS formed during radiolysis. In this context, TiO_2_ can capture secondary electrons produced from water radiolysis or other molecules ionization and participate in surface reactions that prolong the lifetimes of key intermediates. This effect translates into a more sustained generation of oxidative and reductive species, offering advantages in pollutant degradation or microbial inactivation processes [55].

A notable difference between UV and radiation‐driven activation lies in the energy distribution and penetration depth. UV light interacts mainly with the surface of TiO_2_ particles due to limited penetration in turbid media, making photocatalysis highly surface‐dependent. In contrast, ionizing radiation penetrates more deeply into the medium, allowing a more volumetric and homogeneous activation of TiO_2_ particles even in complex aqueous matrices. This volumetric activation can overcome mass transfer limitations observed in conventional photocatalysis and allows efficient treatment of dense suspensions or highly absorbing solutions [56].

Therefore, the role of TiO_2_ under ionizing radiation transcends simple photocatalytic behavior. It becomes an integral component of an advanced oxidation process driven by high‐energy fields, combining radiolytic efficiency with surface catalytic enhancement. This dual function positions TiO_2_ as a valuable material for hybrid advanced oxidation processes (AOPs) aimed at the decomposition of persistent organic pollutants and the mineralization of complex contaminants in aqueous systems.

The reactive species created during irradiation represent the primary agents of chemical change, capable of initiating the degradation of organic compounds in solution. However, the presence of TiO_2_ modifies this radiolytic environment significantly, as previously discus. TiO_2_, enhance the efficiency and selectivity of the degradation process [57, 52, 56]. Under gamma irradiation, TiO_2_ surfaces interact dynamically with radiolytically produced radicals [51]. The oxide surface can act as both a trap and a catalytic site for these reactive species. For example, •OH radicals may adsorb onto the TiO_2_ surface, where they can participate in surface‐bound oxidation reactions with adsorbed pollutants. Simultaneously, conduction band electrons in TiO_2_ may accept hydrated electrons or reduce species such as H_2_O_2_ or O_2_, forming secondary radicals like superoxide (O_2_•^−^) and hydroperoxyl radicals (HO_2_•), which further propagate oxidation cycles [58]. These secondary processes increase the oxidative power of the system beyond what would be achieved by water radiolysis alone.

The synergy between TiO_2_ and radiolysis thus facilitates a more efficient and targeted degradation mechanism. Pollutants near the TiO_2_ surface are subject to both direct oxidation by radicals and indirect degradation through electron–hole processes mediated by the oxide. In particular, hydroxyl radicals generated either in bulk or at the TiO_2_ interface are key contributors to the initial steps of oxidation, breaking down complex organic molecules into smaller intermediates. These intermediates can subsequently undergo further oxidation, often leading to complete mineralization into CO_2_, H_2_O, and inorganic ions [59, 60, 61].

Another important feature is the suppression of recombination reactions. In the absence of catalytic surfaces, reactive radicals can recombine, leading to decreased oxidative efficiency. TiO_2_ helps to stabilize these radicals by facilitating their interaction with pollutants before recombination occurs. Additionally, surface characteristics of TiO_2_, such as crystalline structure, surface area, and presence of oxygen vacancies, can influence the pathway and selectivity of degradation [49, 51].

The implications for contaminant treatment are substantial. Radiolytic systems containing TiO_2_ can efficiently degrade a wide range of recalcitrant pollutants, including pharmaceuticals, pesticides, and industrial dyes, many of which are resistant to conventional treatment methods. The enhanced reactivity and capacity for complete mineralization make TiO_2_ a key component in AOPs driven by ionizing radiation, particularly for applications requiring high efficacy under challenging conditions, such as the treatment of hospital effluents or the remediation of contaminated groundwater [62, 63].

The incorporation of TiO_2_ into radiolytic systems offers several compelling advantages, both from a physicochemical and environmental perspective. One of the most notable attributes of TiO_2_ is its inherently benign nature. As a nontoxic and chemically inert material, TiO_2_ poses minimal risk to human health and the environment, even when applied in large‐scale water treatment scenarios. Its widespread natural occurrence and relative abundance further contribute to its appeal, making it an economically viable option for sustainable water remediation technologies [64]. Equally important is the exceptional radiolytic stability exhibited by TiO_2_. Unlike many organic and inorganic additives that undergo structural degradation or lose activity under high doses of ionizing radiation, TiO_2_ retains its crystalline integrity and catalytic functionality. Studies have shown that both anatase and rutile phases of TiO_2_ can withstand prolonged exposure to gamma irradiation without significant alteration in their surface chemistry or photocatalytic properties. This resilience under harsh conditions ensures long‐term performance and reduces the need for frequent material replacement or regeneration [48, 65].

In terms of treatment efficacy, TiO_2_ contributes to a marked improvement in both dosimetric sensitivity and pollutant degradation. When used in radiolytic systems, TiO_2_ acts as a facilitator of radical‐mediated reactions, effectively enhancing the yield of ROS beyond that produced by water radiolysis alone [59]. The presence of TiO_2_ can shift the balance of chemical pathways, favoring oxidation over recombination, and thereby boosting the radiochemical yield (G‐value) associated with contaminant breakdown. This is particularly beneficial for compounds that are otherwise recalcitrant to radiolysis, as the synergistic action of ROS and surface‐catalyzed mechanisms accelerates their decomposition and mineralization [66]. Moreover, the use of TiO_2_ allows for more efficient utilization of the absorbed radiation dose. By providing catalytic surfaces where radicals can react more selectively and persistently with target molecules, TiO_2_ reduces the energy input required to achieve comparable levels of degradation. This dose efficiency translates into lower operational costs and enhanced scalability, key factors in the deployment of radiolytic water treatment technologies in real‐world applications such as hospital effluent treatment, groundwater remediation, and industrial wastewater management [49].

Altogether, the combination of environmental safety, structural robustness, and catalytic enhancement solidifies TiO_2_ as a cornerstone material in the design of advanced oxidation processes driven by ionizing radiation. Its multifunctional role continues to make it an essential component in emerging treatment systems that aim to address the growing challenge of water contamination in a sustainable and cost‐effective manner.

Despite the many advantages of TiO_2_ in radiolytic systems, its practical application is not without significant limitations. One of the primary concerns involves the limited activation of TiO_2_ under certain radiative conditions. Although TiO_2_ can act as a radiosensitizer, its electron–hole separation efficiency remains a bottleneck, especially in aqueous systems where rapid recombination processes can significantly reduce the yield of ROS necessary for pollutant degradation [67].

Another challenge is related to the surface chemistry and morphology of TiO_2_ particles. The catalytic activity of TiO_2_ is highly dependent on its crystalline phase, surface area, and particle dispersion. In aqueous radiolytic treatments, agglomeration of nanoparticles can occur, which reduces the active surface area and limits mass transfer between the contaminant and the catalyst surface [68]. Moreover, the anatase phase, which is considered more reactive than rutile, is not always thermodynamically stable, particularly under prolonged irradiation or thermal fluctuation, potentially altering the material's effectiveness over time [68, 69].

A further metrological concern arises when assigning absorbed dose in aqueous systems that contain suspended TiO_2_. The Fricke solution is itself the dosimeter and, by definition, should be measured under interference‐free conditions; therefore, the core question is whether Fricke can still provide reliable dose readouts in the presence of TiO_2_ or whether an external reference dosimeter is preferable. TiO_2_ can perturb both the radiation field and the readout pathway: (i) it introduces turbidity and light scattering that bias the spectrophotometric signal at 304 nm, (ii) it may adsorb Fe^2+^/Fe^3+^ or modify radical pathways, altering the effective chemical yield, and (iii) it can change local energy deposition at the particle–liquid interface. Consequently, dose determination in TiO_2_‐loaded media should either (a) decouple Fricke from the catalyst (e.g., co‐irradiated sealed Fricke ampoules, postirradiation particle removal, or optical corrections), or (b) use an independent reference such as alanine pellets to validate or replace Fricke under these conditions [70]. This dual role of TiO_2_ as both catalyst and potential source of measurement error highlights the need for careful system calibration and experimental control.

The synergistic role of TiO_2_ in gamma radiolysis has been explored in various studies targeting emerging pollutants. For instance, several works have documented the effective removal of organic components, where the inclusion of TiO_2_ enhanced both degradation rates and mineralization efficiencies compared to radiolysis alone [55, 71]. Similar enhancements have been reported in the treatment of pharmaceutical compounds like carbamazepine and diclofenac, where TiO_2_ promoted the formation of reactive species and minimized by‐product accumulation [72]. In the case of pesticides, gamma radiation in the presence of TiO_2_ has shown remarkable results in degrading persistent molecule like atrazine, often achieving near‐complete dechlorination under optimized conditions [73]. When comparing these TiO_2_‐assisted systems with those relying solely on ionizing radiation, the catalytic effect is evident: radiolysis alone can achieve significant degradation but often requires higher doses or longer exposure times, while the addition of TiO_2_ allows for lower energy input and faster reaction kinetics [71]. However, it is also worth noting that the catalytic enhancement depends strongly on system configuration, pollutant properties, and water matrix composition.

Interestingly, in many of these studies, TiO_2_ not only serves as a radiosensitizer or photocatalyst but also participates in adsorption processes prior to or during irradiation. This dual function is particularly important when dealing with complex mixtures or trace‐level contaminants, where initial adsorption onto the TiO_2_ surface can increase the local concentration of pollutants near reactive sites, effectively boosting radiolytic degradation [49, 74].

This overlapping role of TiO_2_ highlights a conceptual and practical continuum between catalytic radiolysis and adsorption‐based treatments. In the following section, we delve deeper into the adsorption capacity of TiO_2_ and its role in multicomponent systems, expanding our focus from radiation‐driven degradation to surface‐mediated pollutant removal strategies.

## 4 Interaction of Fricke Solution Components with TiO_2_

The presence of TiO_2_ within irradiated aqueous systems containing Fricke solution raises important questions regarding potential physicochemical interferences. The Fricke dosimeter, based on the radiolytic oxidation of Fe^2+^ to Fe^3+^, is highly sensitive to its chemical environment—particularly pH, the presence of redox‐active species, and competition for reactive radicals [21, 75, 76]. Within this context, TiO_2_ may significantly alter the dynamics of system through metal ion adsorption, shifts in radical distribution, or surface‐mediated reactions that affect dosimetric conversion efficiency.

Previous studies have indicated that both Fe^2+^ and Fe^3+^ can adsorb onto the TiO_2_ surface, especially under acidic conditions similar to those of the Fricke solution (pH < 3) [16]. Although adsorption at such low pH values is generally limited due to the protonation of TiO_2_ surface hydroxyl groups, electrostatic interactions and surface coordination cannot be entirely ruled out. It has been observed that even under highly acidic conditions, the active sites on TiO_2_ may serve as anchoring points for metal species, depending on factors such as ionic strength, competition with other solutes, and the history of the surface material [77, 78].

pH emerges as a key factor governing Fe–TiO_2_ interactions. As the pH increases slightly, the adsorption of Fe^3+^ becomes more favorable due to its higher charge density and smaller ionic radius, which enhance its affinity for TiO_2_ surface oxygen atoms. However, in irradiated systems where TiO_2_ acts as a radiocatalyzer, the adsorption equilibrium is influenced not only by pH but also by the generation of reactive species such as hydroxyl radicals (•OH) and hydrated electrons (e^−^_aq_), which may participate in redox cycling of iron ions [59, 79, 80].

Moreover, the presence of TiO_2_ introduces a competitive dynamic in radical chemistry. Under *γ*‐irradiation, water radiolysis generates reactive species such as •OH, e^−^_aq_, and H•. Several studies have demonstrated that these radicals can interact with the TiO_2_ surface through adsorption and electron transfer processes, instead of reacting directly with Fe^2+^ ions in the bulk solution. In fact, the TiO_2_ surface can trap hydrated electrons (e^−^_aq_) and adsorbed hydroxyl radicals, which may subsequently recombine or participate in redox transformations. Although in classical photocatalysis the generation of electron–hole (e^−^/h^+^) pair requires UV‐light excitation, in radiolytic systems the radical species themselves can inject electrons into the TiO_2_ conduction band or scavenge holes at the valence band, thereby creating surface states analogous to those observed under photonic excitation [49, 81]. This could reduce the dosimetric efficiency of the Fricke system or lead to the formation of secondary products that complicate data interpretation [82, 83]. On the other hand, this interaction might be used to develop hybrid dosimetric systems capable of simultaneously quantifying oxidative and adsorptive processes, although this would require rigorous calibration and standardization protocols not yet established in the literature.

A comprehensive understanding of the interactions between Fricke components and TiO_2_ under irradiation is therefore essential—not only to ensure the accuracy of dose measurements but also to inform the development of integrated technologies for environmental monitoring and treatment.

The interaction between TiO_2_ and the Fricke solution components, particularly Fe^2+^ and Fe^3+^, reveals a complex interplay that cannot be fully understood without considering the underlying adsorption mechanisms. While experimental observations confirm differential affinities and potential interference in radiolytic measurements, a rigorous analysis of the kinetics and equilibrium behavior is essential to quantify these interactions [84]. Moreover, elucidating the adsorption dynamics in both mono‐ and multicomponent systems will allow for a deeper understanding of how TiO_2_ influences the fate of ionic species during irradiation and, consequently, the accuracy of dosimetric outcomes.

## 5 Kinetic and Equilibrium Models of Adsorption

To better characterize the interactions observed in irradiated systems containing TiO_2_ and Fricke solution components, it is crucial to examine adsorption phenomena from a theoretical and quantitative perspective. Adsorption processes are inherently dynamic, involving both temporal evolution (kinetics) and thermodynamic equilibrium states that reflect the affinity between solutes and the solid phase [85, 86]. In this context, adsorption models offer valuable tools to interpret the retention behavior of Fe^2+^ and Fe^3+^ ions on the TiO_2_ surface [87, 88].

This section presents a comprehensive review of kinetic and equilibrium adsorption models relevant to the study of Fe‐based systems. It begins with the analysis of monocomponent systems to establish a foundational understanding of how individual species interact with TiO_2_ [89, 90]. Subsequently, it addresses multicomponent systems where Fe^2+^ and Fe^3+^ coexist and compete for adsorption sites, a scenario that more closely mirrors the conditions of Fricke‐based dosimetry in the presence of TiO_2_ [91]. The discussion culminates in a reflection on how these adsorption behaviors impact dosimetric efficiency and the reliability of chemical dosimeters under modified conditions [92].

### 5.1 Monocomponent Systems

The adsorption of a single solute, such as Fe^2+^ or Fe^3+^, onto TiO_2_ surfaces is typically modeled using well‐established kinetic frameworks. Adsorption kinetics and isotherms can be applied to various systems, with one being of particular interest when TiO_2_ is introduced into the Fricke solution to ascertain the potential for adsorption. It is worth noting that during the oxidation of Fe^2+^ to Fe^3+^ due to the interaction with ionizing radiation, competition for adsorption within TiO_2_ arises, as each ion exhibits a level of adsorption affinity.

Adsorption plays a significant role in a wide range of fields, from surface chemistry and physics to heterogeneous catalysis, water purification, gas adsorption, and interaction of substances in biology [93, 94]. This adsorptive process also involves the retention of contaminants, such as dyes or antibiotics, on the surface of adsorbent material, such as TiO_2_, in order to obtain an aqueous effluent with a lower degree of contamination [95]. A clear assessment of how molecules adhere to a surface is essential for elucidating the role of interfacial interactions in physical and chemical processes. The degree of adsorption can be explored through the utilization of simple models that enable quantitative predictions of how surface coverage varies with pressure, concentration, and temperature [96]. Adsorption is a time‐dependent process. Therefore, it is necessary to determine the adsorption rate to understand the equilibrium time that will occur between the adsorbent and the adsorbate. This adsorbent is typically a solid material that acts as a trap for substances, called adsorbates, present in gases or aqueous solutions [94].

When considering dilute solutions of contaminants, it is assumed that the volume of the solution remains constant during the process. The amount of metal ions adsorbed per unit mass of adsorbent, is quantified using Equation (10) , [95, 97, 98].

(10) $$q=\frac{\left(C_{o-}C_{\mathrm{eq}}\right)*V}{m}$$

where:

*q*: Adsorption capacity (mmol/g)

*C*_o_: Initial concentration of the metal ion in the solution (mM)

*C*_eq_: Equilibrium concentration of the metal ion (mM)

*V*: Volume of the solution (L)

*m*: Mass of the adsorbent (g) [95, 98, 97, 99, 100]

To establish adsorption equilibrium, the adsorbed amount *q* is related to the equilibrium concentration of the solute in the fluid phase (*C*_eq_) through adsorption isotherms (*q* vs. *C*_eq_ at constant T). The determination of both the initial concentration (*C*_o_) and the equilibrium concentration (*C*_eq_) requires accurate analytical methods. In this study, *C*_o_ and *C*_eq_ values for Fe^2+^ and Fe^3+^ were quantified using Standard Method 3500‐Fe B (phenanthroline method) [101] and the colorimetric Fe–thiocyanate method (Fe–SCN) [102], respectively. These datasets form the basis for calibration curves, adsorption kinetics, and adsorption isotherm modeling, which are discussed in the following sections.

Hence, it is essential to determine the adsorption kinetics to derive the phenomenological coefficients that characterize the transport of any adsorbate within a specific adsorbent [94]. The adsorption kinetics of metal ions can be influenced by both the adsorption reaction and mass transfer [97]. To evaluate the experimental data obtained for each metal ion, simplified models of pseudo‐first order or pseudo‐second order are usually applied [95, 94]. Adsorption order refers to the relationship between the concentration of a reactant on the surface of an adsorbent and the rate of the adsorption reaction.

The kinetic models commonly used to predict the adsorption kinetics in any system are detailed in Table 2. In this context, it is important to clarify the meaning of the adsorption capacities used in kinetic models. The term *q*_t_ refers to the amount of solute adsorbed at a given time t (mmol·g^−1^), whereas *q*_e_ represents the adsorption capacity at equilibrium. Both parameters are conceptually consistent with the definition of q presented in Equation (10), which is calculated from the difference between the *C*_o_ and the *C*_eq_ of the solute in solution. Thus, Equation (10) provides the basis for the determination of *q*_e_, while *q*_t_ is obtained experimentally from the concentration of solute at any time t.

**TABLE 2:** Kinetic models.

Name	Model 1	Model 2	Reference
Pseudo‐first order	$\frac{dq_{t}}{dt}=k_{1}(q_{e}-q_{t})$	$q_{t}=q_{e}(1-e^{-k_{1}t})$	[94, 95, 97, 103, 104, 105, 106]
Pseudo‐second order	$\frac{dq_{t}}{dt}=k_{2}{\left(q_{e}-q_{t}\right)}^{2}$	$q_{t}=\frac{k_{2}q_{e}^{2}t}{1+k_{2}q_{e}t}$	[94, 95, 97, 103, 104, 105, 106]

The pseudo‐first order model assumes that the rate‐limiting step in an adsorption process is the mass transfer of the metal ion from the bulk solution to the surface of the adsorbent [97]. In the pseudo‐first order model, rate constant (*k*_1_) is determined, with units of (min^−1^), along with the equilibrium adsorption capacities (*q*_e_) and at a given time (*q*_t_), with units of (mmol/g) [97, 95, 103].

Usually, the pseudo‐second order model is attributed to processes involving a chemisorption mechanism, and it is noteworthy for its ability to estimate the equilibrium loading [95]. In the pseudo‐second order model, the rate constant (*k*_2_) is determined, with units of (g mmol^−1^ min^−1^), along with the equilibrium adsorption capacities (*q*_e_) and at a given time (*q*_t_), with units of (mmol/g) [97, 95, 103].

### 5.2 Equilibrium Adsorption Isotherms (Monocomponent)

Different equilibrium adsorption isotherm models are applied, depending on the specific scenario, in an aqueous solution, containing an adsorbate and a solid which act as an adsorbent. Equilibrium isotherms describe the relationship between the amount of solute adsorbed and its concentration in solution at equilibrium. An aqueous solution containing a single adsorbate interacting with the adsorbent is referred to as a single‐component system [85].

The distribution of Fe^2+^ between solid and liquid phases is of great importance for gaining an understanding of how this ion interacts with TiO_2_ [107]. In such system, the liquid phase, that is, the water solution, will contain an amount of the ion solved, meanwhile the TiO_2_, as a solid phase will have an amount of the same species adsorbed into its surface.

The stages typically involved in the adsorption process, are as follows: (1) transport and diffusion of the adsorbate within the liquid phase to the surface of the solid, (2) surface adsorption of the adsorbate (initial attachment to the solid surface), (3) physical or chemical interactions of the adsorbate with the solid surface (weak Van der Waals forces vs. strong chemical bonding), (4) monolayer or multilayer formation (if applicable) on the adsorbent surface, (5) equilibrium establishment between the amount of the adsorbate present in the solution and the amount of the same substance present in the solid, and (6) desorption (if relevant) of the adsorbate into the liquid phase [108]. The dynamic equilibrium is reached when the rate of adsorption equals the rate of desorption. The adsorbate molecules are in a constant process of attaching to and detaching from the surface of the adsorbent, but the concentration on the surface remained constant [109]. Both linear and nonlinear regression analyses over the equilibrium concentration data have become widely used tools to determine the best‐fitting adsorption models. Both fitting techniques provide insights into the nature and capacity of a specific adsorbent, like the adsorption coefficients and the nature of the adsorption [96, 107, 103].

An isotherm is a graphical representation of the relationship between the amount of adsorbed substance in the solid and its concentration in the liquid phase at equilibrium and at a constant temperature [98, 99]. In the case of gaseous reactants, instead of concentration, the partial pressure of each species is used. Constructing an isotherm involves conducting a series of experiments in which the concentration (or pressure) of the adsorbate in the liquid (or gas) phase is varied, meanwhile the amount of adsorbed substance on the surface of the adsorbent is measured at equilibrium [103, 98]. The specific shape of the isotherm depends on the affinity of the metal for the adsorbent and the adsorption capacity, as well as the experimental conditions present during its construction [98]. This is why each adsorbent and adsorbate have its own characteristics to achieve a specific equilibrium [110]. The adsorption isotherms can also vary to the adsorption heats, which is specific for the adsorbate‐adsorbent pair. Thus, its form is largely determined by the adsorbent characteristics [111].

There are two models for the behavior of adsorbed phase. First, the layer model, which requires the knowledge of the surface area of the solid phase, and second, the pore‐filling model, which is more suitable for porous materials, particularly microporous media, with an uncertain surface area [112]. Commonly used equilibrium adsorption models for describe the layer behavior are the Langmuir and Freundlich isotherms [113, 99, 114]. Meanwhile, models that depict to the pore‐filling mechanism include the Fickian diffusion and surface diffusion [115, 116].

#### 5.2.1 Langmuir Isotherm

Langmuir [117], suggested that the process of adsorption between a liquid with a dissolved adsorbate and a solid acting as an adsorbent is bidirectional, for the adsorbate deposited on the surface could potentially return to the aqueous phase. This model proposes that adsorption occurs on identical and equivalent sites, implying that they have the same probability of being occupied. Consequently, a monolayer is formed without any interactions among the adsorbed molecules, and the maximum adsorption capacity over a homogeneous surface can be estimated [117, 95, 99, 118, 96]. The Langmuir model, stemming from gas‐solid descriptions, is pivotal in quantifying and comparing the adsorbent capacity. This isotherm balances the adsorption and desorption rates at the surface. Adsorption is proportional to exposure, meanwhile desorption is proportional to coverage [107, 110, 119, 100]. The Irving Langmuir isotherm is the simplest and most physically plausible isotherm [96].

The Langmuir Model Is Represented by the Equation (11)

(11) $$q_{e}=\frac{q_{\max}\;K_{L}\;C_{e}}{1+K_{L}\;C_{e}}$$

were:

*q*_e_: Adsorption capacity at equilibrium (mmol/g)

*q*_max_: maximum adsorption capacity (mmol/g)

*K*_L_: Affinity of the adsorbate for the adsorbent (L/mmol)

*C*_e_: Concentration of the solution at equilibrium (mM) [95, 103, 97, 104, 98, 120, 121, 119, 105, 122].

#### 5.2.2 Freundlich Isotherm

The Freundlich model describes the nonideal adsorption between an adsorbate present in an aqueous solution and a solid adsorbent. In this model, the formation of multiple layers on a heterogeneous surface, where the active sites can be distributed exponentially, is assumed [123, 99, 107, 100, 95, 104]. The Freundlich isotherm is characterized by a logarithmic relationship. It aims to account for the interactions between the adsorbed molecules on the surface [96]. One of the most commonly used expressions for the Freundlich isotherm is detailed in Equation (12).

(12) $$q_{e}=K_{F}C_{e}^{\frac{1}{n}}$$

where:

*q*_e_: Adsorption capacity at equilibrium (mmol/g)

*K*_F_: Freundlich constant. Adsorption capacity (mmol/g (mg/L)^1/*n*^)

*C*_e_: Concentration of the solution at equilibrium (mM)

*n*: Adsorption intensity [104, 103, 113, 95, 98, 120, 121, 105, 124]. The magnitude 1/*n* is a measure of the adsorption intensity. directly associated with the efficiency of adsorption, also known as the heterogeneity factor. Values of 0 < 1/*n* < 1 indicate favorable adsorption [99]. Values of *n* are typically less than 1 [97].

### 5.3 Equilibrium Adsorption Isotherms (Multicomponent)

In practical systems, Fe^2+^ and Fe^3+^ coexist and may compete for the same adsorption sites on the TiO_2_ surface. Multicomponent adsorption models are essential for capturing the complexity of such competitive interactions [125]. Conversely, when multiple adsorbates are present in the aqueous solution and come into contact with the adsorbent, a multicomponent system is obtained. If aqueous solutions containing more than one component are available, it is possible to determine the adsorption equilibrium of a specific adsorbent by adjusting the multicomponent isotherms. Through these isotherms, it is possible to deduce the amount of simultaneous removal of these multiple components while also obtaining information about the adsorption extent of the component in the presence or competition with the other species [120].

These multicomponent isotherms also help to clarify the magnitude of competitive anionic interactions in multicomponent mixtures at the solid–liquid interface, understand the behavior of contaminants in the presence of absorption media, and identify the anions affected by other anions. It is therefore necessary to elucidate these complex interactions [126]. Wastewater, on certain occasions, contains more than one component, so it is necessary to study the simultaneous adsorption equilibria of solutions containing two or more solutes [127, 120]. Multicomponent isotherm models extend single‐component adsorption isotherms to multisolute systems, enabling the evaluation of competitive interactions among different adsorbates in aqueous solutions [121, 128].

Table 3 summarizes the main models used to describe adsorption in multicomponent aqueous systems on various adsorbents. The table presents the underlying mechanisms and assumptions of each model, providing insight into how they represent competitive adsorption phenomena. Each model addresses different aspects of the adsorption process, such as site uniformity, surface heterogeneity, and solute–solute interactions, offering a mechanistic understanding of multicomponent adsorption. This information facilitates comparison of the models’ theoretical basis and predictive capabilities, which is crucial for interpreting experimental data and designing adsorption studies in complex systems. Additionally, the scope of study of these models can encompass various fields, such as chemistry, engineering, and environmental science [121, 129].

**TABLE 3:** Equilibrium adsorption isotherm models for multicomponent systems.

Name	Model	Mechanism/Interpretation
Nonmodified Langmuir multicomponent isotherm	$q_{e,j}=\frac{q_{m,i}\;K_{L,i}\;C_{e,i}}{1+\sum_{j=1}^{N}K_{L,j}\;C_{e,j}}$	Assumes monolayer adsorption on uniform sites; multiple solutes compete for the same sites, with adsorption governed by site saturation [121, 129, 130, 131, 132, 133, 134, 135].
Extended freundlich multicomponent isotherm	$q_{e,i}=\frac{K_{F,i}\;C_{e}^{\left(1/ni\right)+x_{i}}}{C_{e,i}^{x_{i}}+y_{i}\;C_{e,j}^{z_{i}}}$	Empirical model describing heterogeneous surface energies; allows nonlinear competition among solutes for adsorption [121, 133, 134, 135, 136].
Extended modified langmuir multicomponent isotherm	$q_{e,j}=\frac{q_{\max,i}\;K_{i}\;C_{i}}{1+\sum_{j=1}^{N}K_{j}\;C_{j}}$	Incorporates modification parameters to account for variations in site affinity; simulates competitive adsorption with adjustable capacity limits [121, 129, 130, 133, 134].
Extended no‐modified langmuir with interaction factor *η* multicomponent isotherm	$q_{e,j}=\frac{q_{m,i}K_{L,i}\left(C_{e,i}/\eta_{i}\right)}{1+\sum_{j=1}^{N}K_{L,j}\left(C_{e,j}/\eta_{j}\right)}$	Incorporates interaction factors (*η*) to model synergistic or antagonistic effects among adsorbates; reflects competition and site influence [121, 129, 131].
Modified Sips isotherm	$q_{e,i}=\frac{q_{\max,i}\;B_{\mathrm{si}}\;C_{\mathrm{ei}}^{n_{si}}}{1+\sum_{j=1}^{N}B_{sj}\;C_{ej}^{n_{sj}}}$	Combines Langmuir and Freundlich behavior; accounts for heterogeneous surface adsorption and competition among solutes [105, 129, 133].
Nonmodified competitive Redlich‐Peterson isotherm	$q_{ei}=\frac{K_{\mathrm{RPi}}\;C_{\mathrm{ei}}}{1+\mathrm{ARPi}\;C_{\mathrm{ei}}^{b_{\mathrm{RPi}}}+\ldots +ARPj\;C_{\mathrm{ej}}^{b_{\mathrm{RPj}}}}$	Empirical model bridging Langmuir and Freundlich; captures nonideal, competitive adsorption behavior for multiple solutes [129, 133, 134, 135].
Modified Redlich‐Peterson Isotherm with Interaction Factor *η*	$q_{ei}=\frac{K_{\mathrm{RPi}}\;C_{\mathrm{ei}}/\eta_{i}}{1+\mathrm{ARPi}\;{\left(C_{\mathrm{ei}}/\eta_{i}\right)}^{b_{RPi}}+\ldots +\mathrm{ARPj}\;{\left(C_{\mathrm{ei}}/\eta_{i}\right)}^{b_{\mathrm{RPj}}}}$	Adds interaction factors (*η*) to describe effects of solute–solute interactions; models competition and modification of adsorption affinity in multicomponent systems [129, 133, 134].

Some Features, Limitations of the Multicomponent Isotherm Models Described in Table 3 Are Outlined below.

#### 5.3.1 Nonmodified Langmuir Multicomponent Isotherm

The nonmodified Langmuir multicomponent isotherm model, developed by Butler and Ockrent [137], assumes independent adsorption of the components. Each component was adsorbed at separate sites without any interactions between them. Among its limitations, it does not account for competitive interactions between components, which could be inappropriate in systems where such interactions are significant. Furthermore, this model may underestimate the actual adsorption in systems with component competition. It is applied in situations where the interactions among the components are insignificant, and it can be assumed that the adsorption of one component does not affect the adsorption of others. It is used to evaluate the adsorption of multiple solutes in systems with minimal or no competitive interactions [130, 121, 134].

#### 5.3.2 Extended Freundlich Multicomponent Isotherm

Sheindrof et al. [136], developed an extended Freundlich‐type multicomponent isotherm model. This model extends the Freundlich model to account for the competitive interactions among the components. It involves coefficients linked to isotherms that can be derived from the mono‐component isotherm data. These coefficients represent the interaction terms that describe how one component affects the adsorption of others [133]. One limitation of this model is that it assumes a linear relationship between the logarithm of the concentration and the logarithm of the adsorbed amount, which may not hold true across all concentration ranges. Furthermore, the interpretation of interaction coefficients can be challenging and may vary with experimental conditions [134]. This model is applicable to systems in which competitive interactions among components are recognized. Therefore, it can be utilized to assess adsorption in aqueous systems with component competition, such as the adsorption of contaminants in wastewater [135, 134].

#### 5.3.3 Extended Modified Langmuir Multi‐Component Isotherm

The extended and modified Langmuir multicomponent isotherm model is an extension of the original Langmuir model, which was adjusted by Jain and Snoeyink [138], to incorporate the interactions among components. Additionally, this model allowed for the determination of the interaction term and affinity of each component toward the active sites of the adsorbent [139, 138]. However, a limitation lies in its ability to fully address the complexities of multicomponent systems with nonlinear interactions despite its focus on competitive interactions. This model finds application in systems where competition among components is suspected to be a significant factor while maintaining the theoretical foundation of the Langmuir model [129, 133].

#### 5.3.4 Extended No‐Modified Langmuir with Interaction Factor *η* Multi‐Component Isotherm

Jain and Snoeyink [140], modified the Langmuir model to incorporate the interaction factor “*η*,” that is a function of sorption capacity [131]. The extended nonmodified Langmuir model with interaction factor *η* multicomponent isotherm represents how interactions between components affect their respective adsorptions. However, a limitation of this model is the complexity in measuring or interpreting the interaction factors “*η*.” This model can be applied in systems where interactions between components with a moderate effect on adsorption need to be considered [121, 129].

#### 5.3.5 Modified Sips Isotherm

The modified Sips Isotherm model combines the elements of the Freundlich and Langmuir models [129]. It is used to describe adsorption in multicomponent systems, particularly in situations where the initial adsorption is rapid and then slows down as the concentration of the components increases. However, its main limitation is that the model parameters may lack direct physical significance and are difficult to interpret. This model is applicable to adsorption systems with an initial rapid growth phase, followed by gradual saturation [105, 133].

#### 5.3.6 Nonmodified Competitive Redlich–Peterson Isotherm

Redlich and Peterson proposed an adsorption isotherm equation [141], which was initially designed for multicomponent systems. The nonmodified competitive Redlich–Peterson isotherm model is an extension of the original model proposed in 1959 and specifically adapted for multicomponent systems involving competition among different components. However, one limitation of this model is that it does not account for the synergistic interactions among the components. This approach is employed to describe and assess adsorption in multicomponent systems where competition exists among components, but it does not address cooperative interactions among them [133, 134].

#### 5.3.7 Modified Redlich–Peterson Isotherm with Interaction Factor *η*

The modified Redlich–Peterson Isotherm model with interaction factor *η* is obtained by modifying the nonmodified competitive Redlich–Peterson Isotherm model with an interaction factor [129]. This modified Redlich‐Peterson model reflects the effect of interactions between the components on adsorption. It is assumed that the surface sites are homogeneous, and that the adsorbate molecules in the aqueous solution compete for these active sites. The limitation of this model is similar to that of the nonmodified Redlich‐Peterson model with the addition of interaction coefficients. The modified Redlich–Peterson isotherm model with interaction factor *η* is used in systems in which the goal is to combine the equilibrium characteristics of the Redlich–Peterson model with the consideration of interactions between components [134, 133].

### 5.4 Implications for Dosimetric Efficiency

Understanding the kinetics and equilibrium of Fe^2+^ and Fe^3+^ adsorption onto TiO_2_ is critical for interpreting Fricke dosimetry in heterogeneous radiolytic systems. Among the potential sources of interference, adsorption of iron species constitutes the most direct mechanism affecting the dosimetric signal, as it alters the effective concentration of redox‐active ions available for radiolytic conversion. Several studies report a preferential affinity of TiO_2_ surfaces for Fe^3+^ species, which can suppress their spectrophotometric detection and lead to an apparent reduction in measured G‐values [142, 143].

In comparison, adsorption of Fe^2+^ plays a secondary but still relevant role. If significant Fe^2+^ sequestration occurs prior to irradiation, the pool of ions available for oxidation is reduced, compromising the fundamental assumption of the Fricke dosimeter that ferrous ions are homogeneously distributed and fully accessible to radiolytic species. This effect may introduce systematic underestimation of absorbed dose, particularly in batch systems where solid–liquid equilibration precedes irradiation.

Beyond adsorption phenomena, additional contributions—such as optical turbidity introduced by suspended TiO_2_ particles or the generation of ROS at the solid–liquid interface—may further perturb dosimetric readings. While turbidity primarily affects absorbance‐based measurements, radiocatalytic pathways could, under certain conditions, modify the effective radiolytic yield by promoting secondary oxidation routes. However, current evidence suggests that adsorption‐driven depletion of Fe^2+^/Fe^3+^ species represents the dominant interference mechanism under strongly acidic Fricke conditions.

Importantly, dosimetric measurements are typically performed postirradiation, whereas adsorption–desorption equilibria may evolve dynamically during irradiation. As a result, postirradiation measurements may not fully capture the transient chemical state of the system under irradiation, introducing additional uncertainty into dose evaluation. These considerations highlight the necessity of explicitly accounting for solid–liquid interactions when applying Fricke dosimetry in TiO_2_‐containing systems.

## 6 Critical Analysis and Research Gaps

Despite considerable advances in understanding the interaction between Fe^2+^/Fe^3+^ species and TiO_2_ surfaces, several critical gaps remain in the literature regarding their implications for chemical dosimetry under irradiation. Experimental studies confirm that TiO_2_ can retain iron species under gamma irradiation, potentially perturbing the redox equilibrium central to Fricke dosimetry [144, 145]. However, most investigations have been conducted under nonirradiated conditions or have focused on isolated parameters such as pH, contact time, or initial concentration, without fully accounting for the coupled dynamics of adsorption and radiolysis characteristic of irradiated systems [146].

A particularly critical issue concerns the physicochemical interpretation of adsorption under strongly acidic Fricke conditions (pH < 1). Such acidic environments are intentionally employed to stabilize iron predominantly in its ferric state (Fe^3+^), ensuring the reliability and linearity of the Fricke dosimetric response. Under these conditions, TiO_2_ surfaces are predominantly protonated, and simple electrostatic attraction of cationic species is indeed unlikely. However, this does not preclude adsorption altogether. Instead, adsorption of Fe^2+^ and especially Fe^3+^ must be interpreted in terms of specific inner‐sphere surface complexation, coordination interactions, or chemisorption mechanisms, rather than classical electrostatic attraction [147, 148].

In acidic Fe–Ti–O systems, Fe (III) is known to form stable coordination environments with oxygenated surfaces and mixed metal–oxide frameworks. Iron can interact directly with surface titanium–oxygen groups, leading to the formation of Fe–O–Ti surface linkages or mixed environments analogous to ilmenite‐like (FeTiO_3_) or iron–titanate structures, which are well documented in mineralogical, synthetic, and composite systems [149, 150]. Importantly, such interactions are governed by chemical affinity and orbital coordination rather than by the net surface charge, allowing adsorption and surface retention of Fe (III) even under strongly acidic conditions.

These chemically specific interactions are consistent with reports describing Fe (III)–TiO_2_ composite formation, iron‐doped titanium systems, and iron recovery or separation challenges in Ti‐containing acidic matrices, highlighting the strong chemical association between Fe and Ti oxides under low‐pH conditions [151, 152]. Despite this, these mechanisms are rarely quantified or incorporated into dosimetric models, particularly under irradiation conditions. This lack of mechanistic integration represents a significant source of uncertainty when interpreting Fricke dosimetry in heterogeneous radiolytic systems [153].

Another major limitation is the lack of standardized methodologies to assess the dosimetric reliability of Fricke solution in systems containing solid phases such as TiO_2_. While kinetic and equilibrium models—including Langmuir, Freundlich, and pseudo‐second‐order formulations—have been widely applied to describe ion retention [86, 85], there is no consensus on their validity under irradiation conditions. In particular, competitive adsorption in multicomponent systems, where Fe^2+^ and Fe^3+^ coexist with radiolytic intermediates, is frequently overlooked despite its direct relevance to real dosimetric environments [154].

The reversibility of adsorption processes under irradiation further complicates interpretation. If adsorption–desorption equilibria were to shift dynamically during irradiation, postirradiation measurements might not accurately reflect the chemical state governing dose deposition. However, under strongly acidic and controlled pH conditions characteristic of Fricke dosimetry, spontaneous desorption is not expected unless the chemical environment is altered. Since *γ*‐irradiation does not induce structural or compositional modifications of TiO_2_ or iron oxide phases, adsorption–desorption behavior remains governed by coordination and surface complexation equilibria rather than by radiation‐induced surface alteration [155, 156].

At the same time, it remains unresolved whether TiO_2_ acts solely as a passive adsorbent or whether effective energy transfer and radiocatalytic processes contribute to modifications of the intrinsic radiolytic yield (G‐value) of the Fricke system. Radiocatalytic activity of TiO_2_ under *γ*‐irradiation has been reported, supporting the possibility that adsorption‐driven interference and genuine modifications of radiation chemistry may coexist [52]. This ambiguity limits the ability to unambiguously distinguish adsorption effects from radiocatalytic contributions in modified dosimetric systems.

Future research should therefore focus on integrative approaches that couple adsorption modeling with radiation chemistry, explicitly addressing time‐dependent effects and solid–liquid interactions. Standardization efforts should define experimental protocols capable of distinguishing adsorption‐driven interference from genuine radiolytic enhancement while systematically evaluating the influence of dose, TiO_2_ loading, ionic strength, and matrix composition [157]. Such efforts are essential to ensure accurate dose measurement in heterogeneous radiolytic systems and to advance the reliable use of chemical dosimetry in radiocatalytic applications.

From an operational perspective, additional hurdles must also be considered. The separation and recovery of TiO_2_ after treatment remain challenging: while immobilization techniques (e.g., coating on supports) can help retain the catalyst within the system, they may also hinder surface reactivity and mass transfer. Conversely, the use of TiO_2_ in colloidal form requires subsequent filtration or sedimentation steps, increasing both cost and operational complexity [158]. Moreover, the influence of water matrix constituents—such as natural organic matter, carbonate ions, and salts—can inhibit TiO_2_ or other semiconductors (e.g., ZnO) by competing for reactive sites or altering surface charge properties. These interactions reduce the availability of ROS for pollutant degradation and highlight the necessity of considering environmental factors when assessing TiO_2_‐based radiolytic systems [61].

Overall, this critical analysis underscores the urgent need to bridge the gap between adsorption science and radiation chemistry in the context of chemical dosimetry. Addressing these gaps is essential not only for refining existing dosimetric models but also for ensuring accurate dose measurements in advanced water treatment systems involving radiocatalysis. These considerations naturally lead to a deeper evaluation of how TiO_2_‐induced adsorption phenomena ultimately influence the dosimetric efficiency of the Fricke system.

## 7 Summary and Outlook

This review has explored the complementary roles of Fricke dosimetry and titanium dioxide (TiO_2_)‐based adsorption in the field of radiolytic systems, with a focus on their integration in aqueous‐phase treatment and chemical analysis. The Fricke solution remains a reference standard in chemical dosimetry due to its well‐characterized redox behavior and linear dose–response under controlled conditions. Meanwhile, TiO_2_, through its high surface reactivity and photocatalytic and radiosensitizing properties, has demonstrated great potential in advanced oxidation processes and pollutant degradation under ionizing radiation.

An emerging area of interest is the interaction between these two systems under radiolytic conditions. Experimental evidence suggests that TiO_2_ can influence the radiolytic yield of ferrous ions in Fricke solutions, either by adsorbing Fe^2+^/Fe^3+^ species or altering the local distribution of ROS, thereby modifying the dosimetric response. Although the extent of these interactions varies depending on system parameters such as pH, dose rate, and TiO_2_ surface characteristics, the implications for both dosimetry and water treatment processes are significant.

Despite these insights, current research remains fragmented, with limited studies exploring the mechanistic overlap between adsorption, radiolysis, and redox dosimetry. The lack of standardized protocols for hybrid systems combining TiO_2_ and Fricke solutions presents challenges in reproducibility and interpretation. Furthermore, the role of multicomponent matrices, often present in real effluents, is insufficiently studied in relation to competitive adsorption and its effect on dosimetric linearity.

Looking forward, there is a strong case for developing integrated analytical frameworks that consider both chemical dosimetry and adsorption dynamics in radiolytic environments. Future work should aim to (1) delineate the mechanistic pathways by which TiO_2_ influences Fricke dosimetry, (2) establish kinetic and equilibrium models for Fe^2+^/Fe^3+^ interactions with TiO_2_, and (3) assess the dosimetric accuracy of Fricke systems in complex, multicomponent aqueous media. The advancement of such approaches could significantly enhance the reliability of radiation‐based treatments and the precision of absorbed dose calculations in heterogeneous systems.

## Funding

This work was supported by Escuela Politécnica Nacional (PII‐DCN‐2023‐01).

## Conflicts of Interest

The authors declare no conflicts of interest.
